# Optimal application of stereotactic body radiotherapy and radiofrequency ablation treatment for different multifocal hepatocellular carcinoma lesions in patients with Barcelona Clinic Liver Cancer stage A4–B1: a pilot study

**DOI:** 10.1186/s12885-021-08897-z

**Published:** 2021-10-30

**Authors:** Feiqian Wang, Kazushi Numata, Atsuya Takeda, Katsuaki Ogushi, Hiroyuki Fukuda, Hiromi Nihonmatsu, Koji Hara, Makoto Chuma, Yuichirou Tsurugai, Shin Maeda

**Affiliations:** 1 Ultrasound Department, The First Affiliated Hospital of Xi’an Jiaotong University, No. 277 West Yanta Road, Xi’an, Shaanxi, 710061 People’s Republic of China; 2 Gastroenterological Center, Yokohama City University Medical Center, 4-57 Urafune-cho, Minami-ku, Yokohama, Kanagawa 232-0024 Japan; 3 Radiation Oncology Center, Ofuna Chuo Hospital, Kamakura, Kanagawa 247-0056 Japan; 4 Division of Gastroenterology, Yokohama City University Graduate School of Medicine, 3-9 Fukuura, Kanazawa-ku, Yokohama, Kanagawa 236-0004 Japan

**Keywords:** Radiofrequency ablation, Stereotactic body radiotherapy, Hepatocellular carcinoma, Tumor response, Recurrence, Survival

## Abstract

**Background:**

In clinical practice, many hepatocellular carcinoma (HCC) patients in Barcelona Clinical Liver Cancer (BCLC) stage A4–B1 cannot receive the curative treatments of liver transplantation, resection, and radiofrequency ablation (RFA), which are the recommended options according to liver cancer guidelines. Our aim is to study the feasibility of RFA and stereotactic body radiotherapy (SBRT) as a curative treatment for different multifocal HCCs in BCLC stage A4–B1 patients.

**Methods:**

From September 2014 to August 2019, 39 multifocal HCC lesions (median diameter: 16.6 mm) from 15 patients (median age: 73 years) were retrospectively selected. Among them, 23 were treated by RFA and the other 16 by SBRT because of predictable insufficiency and/or risk related to RFA performance. The indicators for evaluating this novel therapy were the tumor response, prognosis (recurrence and survival), and adverse effects (deterioration of laboratory test values and severe complications).

**Results:**

The median follow-up duration was 31.3 months (range: 15.1–71.9 months). The total patients with a one-year complete response, stable disease, or disease progression were 11, 1, and 3, respectively. In total, 8 and 2 patients had confronted intrahepatic or local recurrence, respectively. The one-year progression-free survival rate and local control rate were 80% (12/15 patients) and 97.4% (38/39 lesions), respectively. The median time to progression was 20.1 (2.8–45.1) months. The one- and two-year survival rates were 100 and 88.9%, respectively. In up to five months’ observation, no patient showed severe complications. Seven, four, and two patients had slight changes in their white blood cells, platelet count, or albumin–bilirubin grade, respectively.

**Conclusions:**

For patients with BCLC stage A4–B1, RFA and SBRT treatment for different multifocal HCCs may be a potential option because of the favorable prognosis and safety. However, before its application in clinical practice, prospective, controlled, large-scale studies are needed to further confirm our conclusions.

## Background

Hepatocellular carcinoma (HCC) is a heterogeneous disease. The management of the therapeutic approach to its treatment may vary and strongly relates to the patients’ states, the characteristics of lesions, and the tumor stage. According to the most commonly used staging system, Barcelona Clinical Liver Cancer (BCLC) [1, 2], and the recently developed Kinki Criteria [3], BCLC stage A4 is defined as a patient in Child-Pugh A–B, presenting with three nodules ≤3 cm, while stage B1 in Child-Pugh scores 5–7, has multinodules beyond Milan, and falls within the up-to-7 standard. In short, BCLC stages A4–B1 are characterized as patients with relatively good liver function and multiple, small lesions, but neither portal invasion nor extrahepatic spread. With these favorable characteristics, they have the opportunity to receive potentially curative treatment [4] rather than palliative treatment. As patients in BCLC stage B1 have a comparable prognosis (overall survival and disease-free survival) to those in BCLC stage A, a recent study suggested that BCLC stage B1 patients be re-classified into early stage [5]. Percutaneous image-guided radiofrequency ablation (RFA) is a recommended therapy for early-stage HCC given its advantages of minimal invasion, promising long-term survival, and up to 90% local tumor control for small-sized HCCs [6, 7]. However, in daily clinical practice, RFA performance is sometimes dangerous, insufficient, or even unfeasible due to anatomical factors (e.g., diameter > 3 cm, adjacent to the liver capsule, main vessels or gastrointestinal tract, deep-located near dome or subphrenic area) and technical ultrasound (US) factors involving poor conspicuity [8, 9]. In particular, perivascular lesions (though maybe small in size) inevitably produce the heat-sink effect, which may result in residue.

Stereotactic body radiotherapy (SBRT) is an emerging non-surgical locoregional treatment modality that involves the delivery of high individual doses of radiation with high geometric precision and accuracy to the targeted lesion [10]. Though not recommended in the latest international guideline to treat early-stage HCC [11, 12], SBRT is suggested in many research papers as a promising treatment option for early-stage and small HCCs because of its minimal blood vessel, bile duct, gastrointestinal, and hepatocyte toxicities, along with statistically favorable local control rates, prolonged progression-free survival (PFS), and improved overall survival (OS) [7, 13–16]. Specifically, for the patient population with a mean Child–Pugh score of 6.4 (A5–C11) and HCC lesions with mean size of 2.7 cm (1.1–5.6 cm), the reported local control rate, PFS, and OS in one year using SBRT treatment are 95, 66, and 87%, respectively [17]. When compared to RFA treatment in patients with similar basic characteristics, SBRT treatment exhibits excellent local control (the two-year local control rate was 83.8% after SBRT and 71.8–80.2% after RFA) and comparable OS (the one- and two-year OS rates were 70 and 53% after RFA, and 74 and 46% after SBRT, respectively) [18–20]. By comparative analysis of a Markov model, Seo et al. [7] proposed SBRT as the preferred treatment option over RFA for 2–3 cm HCC lesions. Recently, to overcome the limitations of insufficient ablation in certain locations, an SBRT method to treat post-ablative local progression has been developed. Good results for the one-year local control rate (81.8–86.6%), PFS (63.3–69.9%), and OS (85.4–85.6%) have been reported [21]. To treat local recurrence of initial RFA, SBRT is considered to be comparable and more cost-effective than repeated RFA [22], especially for patients with larger tumors or tumors abutting major vessels [23]. In summary, SBRT has shown good results at improving the patient prognosis, both when compared to the recommended treatment option, RFA, and when used in a compensatory manner after RFA.

Inspired by the previously successful combined application of RFA and SBRT in HCC patients at a relative early stage [21, 22], and considering the requirement for an effective treatment strategy for early multifocal HCCs, especially those ineligible for RFA performance or without it available, we propose a novel treatment strategy combining SBRT and RFA for different HCC lesions coexisting in the same patient. We performed a retrospective single-arm study by applying RFA and SBRT treatment to 39 multifocal HCC lesions from 15 patients in BCLC stage A4–B1, evaluating the benefit for the prognosis and possible adverse effects during follow-up, then determining the comprehensive therapeutic consequences. We expect this novel strategy to be feasible and effective, and thus it could present be an alternative curative strategy for multiple small lesions in patients with good liver function.

## Methods

### Patient selection

From September 2014 to February 2019, 294 consecutive patients with a total of 480 HCC lesions, which were treated by RFA or SBRT, were initially selected. All patients were local and Japanese and had been diagnosed with HCC at Yokohama City University Medical Center (YCUMC). HCC was diagnosed according to the Japan Society of Hepatology Guideline [24]; these lesions all had a typical imaging appearance of HCC and/or HCC was histologically proven by biopsy. All RFA was performed at YCUMC, while SBRT treatment was provided at Ofuna Chuo Hospital. YCUMC had complete medical records for all patients as the first diagnoses of HCC and follow-up of these patients were conducted at YCUMC. The data collection and analysis received institutional review board approval at YCUMC, and written informed consent was waived due to the retrospective observational nature of the study.

The inclusion criteria were patients (1) with tumors diagnosed as multifocal HCC at stage A4 or B1, for which they had initially received treatment; (2) for whom undergoing surgery was found to be unfeasible, difficult, or unsuitable (the reasons for inoperability included a central location, decreased liver function, and other comorbidities); (3) for whom the multifocal lesions were initially treated by either RFA or SBRT; and (4) treated at intervals between the RFA and SBRT of no more than three months.

The exclusion criteria were as follows: (1) patients in BCLC stages 0–A3 (170 patients with 170 lesions); (2) patients in BCLC stages greater than B1 (9 patients with 30 lesions); (3) patients who the hospital lost contact with prior to one year’s follow-up (13 patients with 30 lesions); (4) patients unable to meet the modified Response Evaluation Criteria in Solid Tumors (mRECIST) [25] for the selection of target lesions (less than two lesions ≥1 cm and failed to show pre-treatment enhancement on arterial phase (AP) of computer tomography (CT) or magnetic resonance imaging (MRI) examination; 37 patients with 79 lesions); (5) patients whose multifocal lesions were initially treated in the same way (i.e., all by RFA or all by SBRT; 46 patients with 123 lesions); and (6) patients with treatment intervals between the RFA and SBRT of more than three months (four patients with nine lesions).

### Treatment strategy

#### RFA procedure

As described by Wang et al. [26] and Hao et al. [27], a 480 kHz generator (VIVA RF generator; STARmed, Gyeonggi, Korea) capable of producing a maximum power of 200 W and a specifically sized 17-gauge internally cooled, adjustable RF electrode (Proteus; STARmed, Gyeonggi, Korea) were applied. RFA was performed with the guidance of real-time CT/MRI and US fusion imaging carried out by one of the three senior hepatologists (K.N., K.O., and H.F.), each having more than 20 years of experience in interventional techniques. One to three insertions were performed according to the tumor size and shape, requiring an ablative safety margin of no less than 5 mm around the treated lesions. Post-operative contrast-enhanced ultrasonography was performed to determine the adequacy of ablation. If a residual tumor was detected, additional RFA was performed.

#### SBRT procedure

We previously described our SBRT methods in detail [28]. During free breathing of patients, Spiral, 4-phase, multidetector CT and/or dynamic contrast-enhanced MRI were conducted, and followed by fusion with a slow-scan CT scan (6–10 s per slice). The gross tumor volume (GTV), including the enhanced tumor, was delineated with the slow-scan CT images. For the internal target volume, an internal margin (4–6 mm) was created around the clinical target volume (CTV) according to the respiratory movement of the diaphragm observed during fluoroscopy. For the planning target volume (PTV), individualized margins of 2 mm were applied around the internal target volume as a setup margin. Multiarc, dynamic conformal radiation was planned using a radiation treatment planning system (FOCUS XiO, version 4.2.0–4.3.3; Computerized Medical Systems, St Louis, MO, USA) and was performed using X-rays from a 6-MV linear accelerator (Clinac 2100C; Varian Medical Systems Inc., Palo Alto, CA, USA). When the tumors were not near the gastrointestinal tract, SBRT with total doses of 35–40 Gy were delivered in five fractions over five to seven days. The total dose of 35 Gy was administered to patients with Child–Pugh class A or B disease, with > 20% of the normal liver receiving > 20 Gy, and a total dose of 40 Gy in the other patients. For the tumors near the gastrointestinal tract (≤ 3 mm distance), a total dose of 42 Gy was delivered in 14 fractions over 18 days [16]. The treatment was planned to enclose the planning target volume with a 60–80% isodose line of the maximal dose.

### Interpretation and assessment of outcome data

#### Adverse effects

The first targets of observation in this study were treatment-related adverse effects, which were evaluated by both laboratory testing (objective findings) and clinically-visible complications (constitutional symptoms) [29]. The laboratory parameters, including serum alanine transaminase (ALT), aspartate transaminase (AST), leukocytes counts, platelets counts, total bilirubin (T-BIL), and albumin (ALB), were recorded. Hematologic toxicity by radiation, a principal cause of acute myelosuppression, was evaluated by the decline in leukocytes and platelets. The albumin–bilirubin (ALBI) score was calculated according to the values of T-BIL and ALB. The ALBI grade, ALT, and AST were used to assess treatment-related side effects on liver function. The time points for laboratory evaluations were one or three days before treatment, one week after completion of later therapy (either RFA or SBRT), and three to five months thereafter. In addition, clinically-visible complications were recorded and graded according to the National Cancer Institute Common Terminology Criteria for Adverse Events version 4.03. The adverse events grade ≥ 3 were recorded for later analysis.

#### Tumor response and prognosis

The secondary targets for observation were tumor response and local control. Tumor response was evaluated one year after treatment completion of both RFA and SBRT. According to mRECIST for patients with multifocal HCC lesions, the longest viable tumor diameter of the two selected target lesions was measured [30]. Local control was assessed by follow-up radiologic imaging and defined as no tumor recurrence/progression at the primary site.

The tertiary observation target was the prognosis, including time to progression, one- and two-year PFS, and OS. PFS was defined as the time from the initial treatment (RFA or SBRT in our study) of the target HCC lesion to the first occurrence of disease progression or death from any cause, whichever occurred first. OS was defined as the interval from the initially received therapy (either RFA or SBRT) until the last visit or date of death, regardless of the cause of death. The outcome of this study was death or dropout of the patients. Exposure factors were RFA and SBRT treatment. Potential confounders that influence OS include other chronic diseases (diabetes, hypertension, etc.) that the patient also suffers from. They were calculated from the date of the earlier therapy (either RFA or SBRT). Local control was evaluated on a lesion basis; the tumor response, PFS, and OS were evaluated on a patient rather than lesion basis.

#### Attention points for imaging evaluation

Contrast-enhanced CT and MRI were used as first-line imaging modalities for evaluating tumor response and recurrence in all patients. Contrast-enhanced ultrasound examination and non-enhanced CT or non-enhanced MRI were used for one patient experiencing renal dysfunction during the follow-up periods. Radiological images were all independently evaluated by two hepatologists (enhanced CT and MRI: M.C. and K.H.; contrast-enhanced ultrasound: H.N. and K.O.) who were unaware of any laboratory test information, clinical history, or our therapy strategy. Any interpretation discrepancies were resolved by consensus with the participation of a third expert hepatologist (K.N.) with 20 years of experience in HCC diagnosis and treatment.

### Statistical analysis

According to type and level of distribution, the continuous variables (lesion size, treatment intervals, PFS, and OS) are presented as means and ranges while the categorical variables (such as treatment modalities and rise or fall of laboratory test values) are described as percentages and frequencies, as appropriate. Cumulative rates of PFS and OS were estimated using the Kaplan–Meier method. Analyses were performed using the statistical software SPSS 24.0 (Inc, Chicago, IL). All analyses are descriptive.

## Results

### Selected patients

**Fig. 1:**
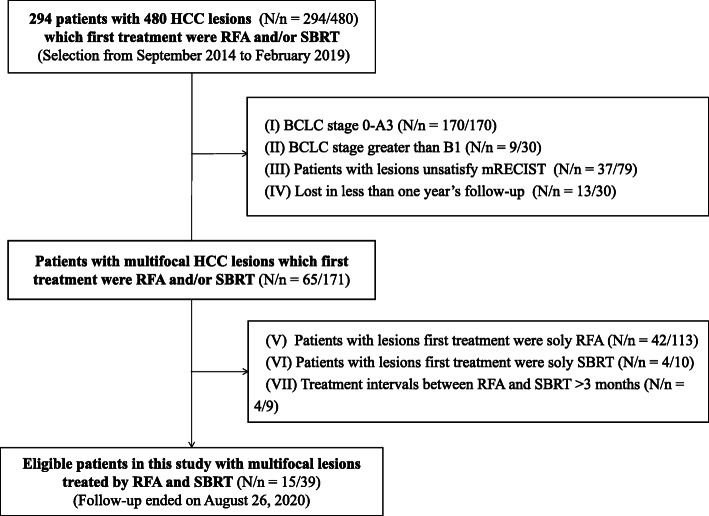
Flowchart of the study population. In total, 39 and 16 lesions and patients were finally used for data analysis, respectively. One exclusion criterion (III) indicates that the patients all had small lesions (fewer than two lesions ≥1 cm) and failed to show pre-treatment enhancement on the arterial phase of CT or MRI examination. Abbreviations: HCC: hepatocellular carcinoma; RFA: radiofrequency ablation; SBRT: stereotactic body radiotherapy; BCLC:Barcelona Clinical Liver Cancer; mRECIST: modified Response Evaluation Criteria in Solid Tumors

Fifteen patients were eligible for this study. The study population selection is presented in Fig. 1. Twelve of the patients initially received RFA therapy and subsequently SBRT, whereas the other three patients first received SBRT treatment and then RFA. There was no strict guideline followed for the order of these two therapeutic approaches. The treatment order depended on the available schedules of patients and doctors with a consensus. The median treatment interval of SBRT and RFA treatment was 26 (4–60) days.

The baseline characteristics of the selected patients and lesions are summarized in Tables 1 and 2, respectively. Most of the lesions treated by SBRT in our study were located in segment seven or eight, which are blind areas hidden by the lung gas during the patient’s deep breathing (nine cases). Other conditions included lesions adjacent to the main portal or hepatic vein (three cases), located close to the heart (two cases), or with poor conspicuity in US and contrast-enhanced ultrasound images (one case).

**Table 1:** Baseline characteristics of enrolled patients /lesions^1^

Patient No.	No. lesions	Age (years)	Etiology	Child–Pugh grade	ALBI grade	BCLC stage	AFP (ng/ml)	ALB (g/dL)
1	2	60s	HCV	A6	2	B1	153	3.3
2	2	80s	HCV	A6	2	A4	56	3.2
3	2	60s	HCV	A5	1	B1	581	4.6
4	5	60s	HCV	A5	1	B1	2	4.1
5	2	60s	HCV	A5	2	A4	9	4.0
6	3	80s	Alc	A5	1	B1	62	4.6
7	2	60s	HCV	A6	2	A4	144	3.4
8	4	70s	HCV	A5	2	B1	11	3.5
9	4	70s	NBNC	A5	1	B1	4	4.3
10	2	80s	HCV	A5	2	A4	9	3.9
11	2	70s	HCV	A5	1	A4	7	4.7
12	2	80s	HCV	A6	1	B1	66	4.4
13	2	80s	HCV	B7	1	A4	12	4.7
14	3	80s	NBNC	A5	2	A4	4	4.0
15	2	60s	HCV	A5	1	A4	2	4.9
Total^2^	2.7 (2–5)	73 (63–86)	12/2/1	9/4/1	8/7	8/7	73.2 (2–581)	4.1 (3.2–4.7)

^1^ HCC: Hepatocellular carcinoma; HCV: hepatitis C virus; HBV: hepatitis B virus; NBNC: non-HBV non-HCV; Alc: Alcohol abuse; BCLC: Barcelona Clinic Liver Cancer; ALBI:albumin–bilirubin; AFP: alpha-fetoprotein; ALB: Albumin; M:male; F:female

^2^ In this line, the order for etiology is HCV, NBNC, and Alc; for Child–Pugh grade, the order is A5, A6, and B8; while for BCLC stage, it is A4 and B1

**Table 2:** Baseline characteristics of RFA and SBRT treatment on different multifocal lesions^1^

Patient No.	Lesion No.	Size (mm)	Segmental location	Treatment modalities	Reason for choosing SBRT
1	1	11	5	RFA	Close to diaphragm and hepatic vein
	2	40	7	SBRT	
2	3	22	2	RFA	Close to dome of diaphragm
	4	25	8	SBRT	
3	5	18	7	RFA	Adjacent to portal vein
	6	33	8	SBRT	
4	7	13	8	RFA	Unavoidable hepatic vein (> 3 mm in diameter) in puncture path
	8	11	8	RFA	
	9	7	8	RFA	
	10	14	1	SBRT	
	11	11	1	SBRT	
5	12	10	5	RFA	Adjacent to portal vein
	13	14	2	SBRT	
6	14	12	8	RFA	Large size and close to dome
	15	12	8	RFA	
	16	40	8	SBRT	
7	17	18	6	RFA	Close to dome of diaphragm
	18	20	4	SBRT	
8	19	18	3	RFA	5 mm distance from heart
	20	9	8	RFA	
	21	10	8	RFA	
	22	17	4	SBRT	
9	23	20	8	RFA	Close to dome of diaphragm
	24	10	4	RFA	
	25	8	8	RFA	
	26	11	8	SBRT	
10	27	21	3	RFA	Close to dome of diaphragm
	28	17	7	SBRT	
11	29	16	8	RFA	Undetectable in US and unclear in CEUS
	30	22	7	SBRT	
12	31	11	6	RFA	Close to dome of diaphragm
	32	32	8	SBRT	
13	33	10	7	RFA	Close to dome of diaphragm
	34	17	7	SBRT	
14	35	17	8	RFA	Close to dome of diaphragm
	36	17	8	RFA	
	37	8	4	SBRT	
15	38	15	6	RFA	Close to heart
	39	10	3	SBRT	
Total ^2^	/	16.6 (7–40)	2/2/3/4/2/3/6/17	23/16	/

^1^ HCC: Hepatocellular carcinoma; RFA: radiofrequency ablation; SBRT: stereotactic body radiotherapy; US: ultrasound; CEUS: contrast-enhanced US

^2^ In this line, the order of segmental location is 1 to 8. The order of treatment modalities was RFA and SBRT. The value of size is displayed as mean and range

### Tumor response and prognosis

**Fig. 2:**
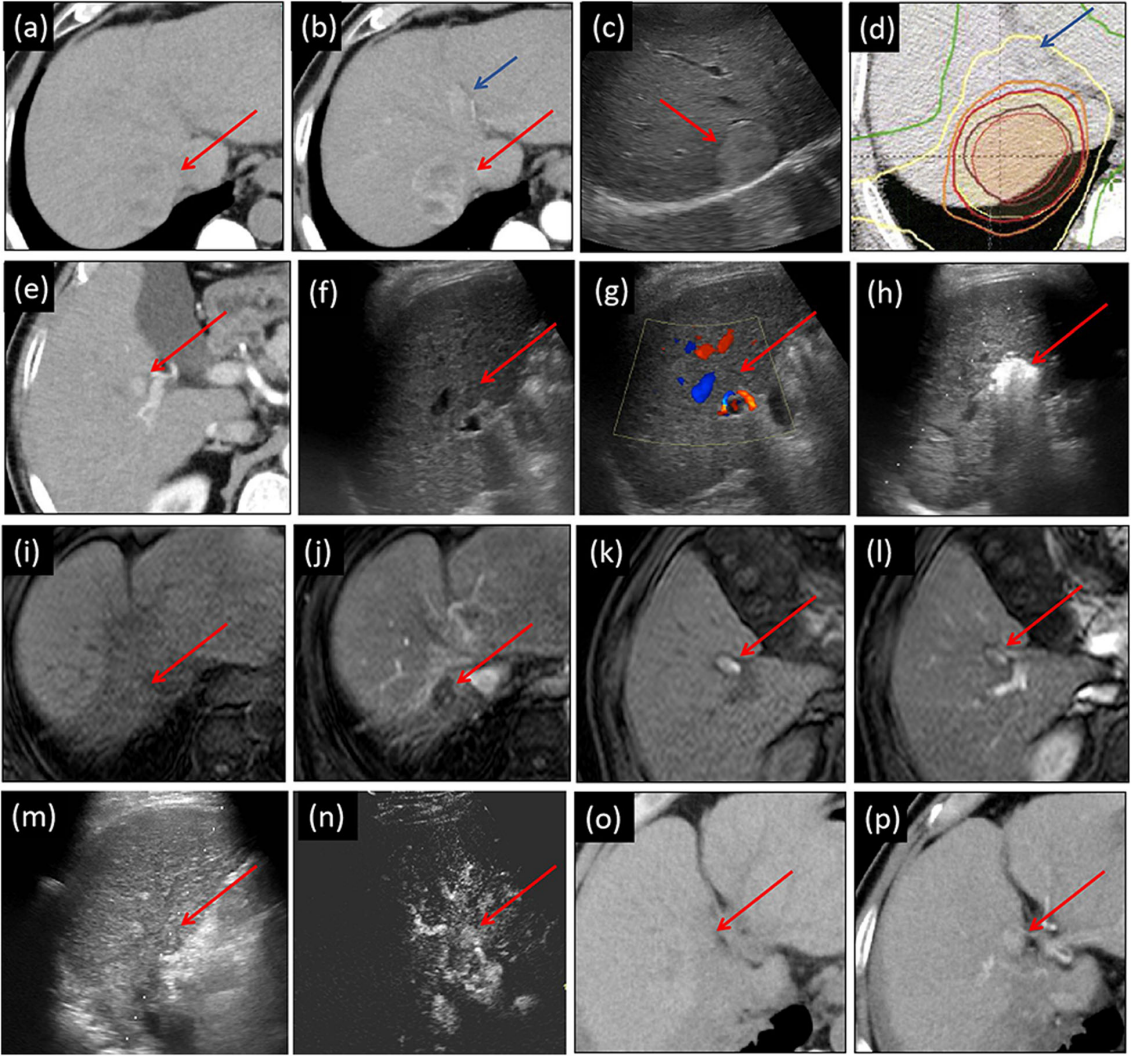
A case of complete tumor response and intrahepatic distal recurrence. (**a,b**) A 40 mm lesion in segment (S)7 was planned for SBRT, showing unenhanced CT and arterial phase (AP) of contrast-enhanced CT images, respectively. The lesion was partial hyperenhanced. (**c**) Grayscale ultrasound (US) image shows the lesion in S7 located adjacent to the diaphragm and hepatic vein. It was estimated that ablation would be risky because of its location. The lesion was well-defined and with a nodule-in-nodule appearance. (**d**) A dose distribution picture of SBRT treatment plan was generated. A total dose of 40 Gy (red isodose line) was delivered in 5 fractions. The central part received 55 Gy radiation. (**e**) An 11 mm lesion located in S5 was detected in the AP of the contrast-enhanced CT image. (**f,g**) Grayscale US and color Doppler flow image of the lesion. (**h**) Grayscale US image in the process of RFA. (**i,j**) Compared with unenhanced T1-weighted MR image (i), at the one-year follow-up, the SBRT-treated area considerably decreased and changed into totally hypoenhancement (**j**). (**k,l**) At the one-year follow-up, the RFA-treated area changed into hyperenhanced scars in both the unenhanced T1-weighted image (**k**) and the AP of Gadolinium-Ethoxybenzyl-Diethylenetriamine Pentaacetic Acid MRI (EOB-MRI) (**l**). (**m,n**) After 44.8 months’ follow-up, a new 10 mm lesion was detected in S4. In grayscale US (m), it appeared as slightly hyperechoic and poorly defined. In the AP of contrast-enhanced US (n), the lesion displayed hypervascularity. In the unenhanced CT(o) taken as a reference, the AP of the contrast-enhanced CT image (p) showed hyperenhancement. Red arrows in (a–c) and (e–p) indicate the location of the target lesion or post-treated area. Dark blue arrows in (b) and (d) show the approximate position of the S7 lesion. This case corresponds to the No. 1 patient shown in the tables

**Fig. 3:**
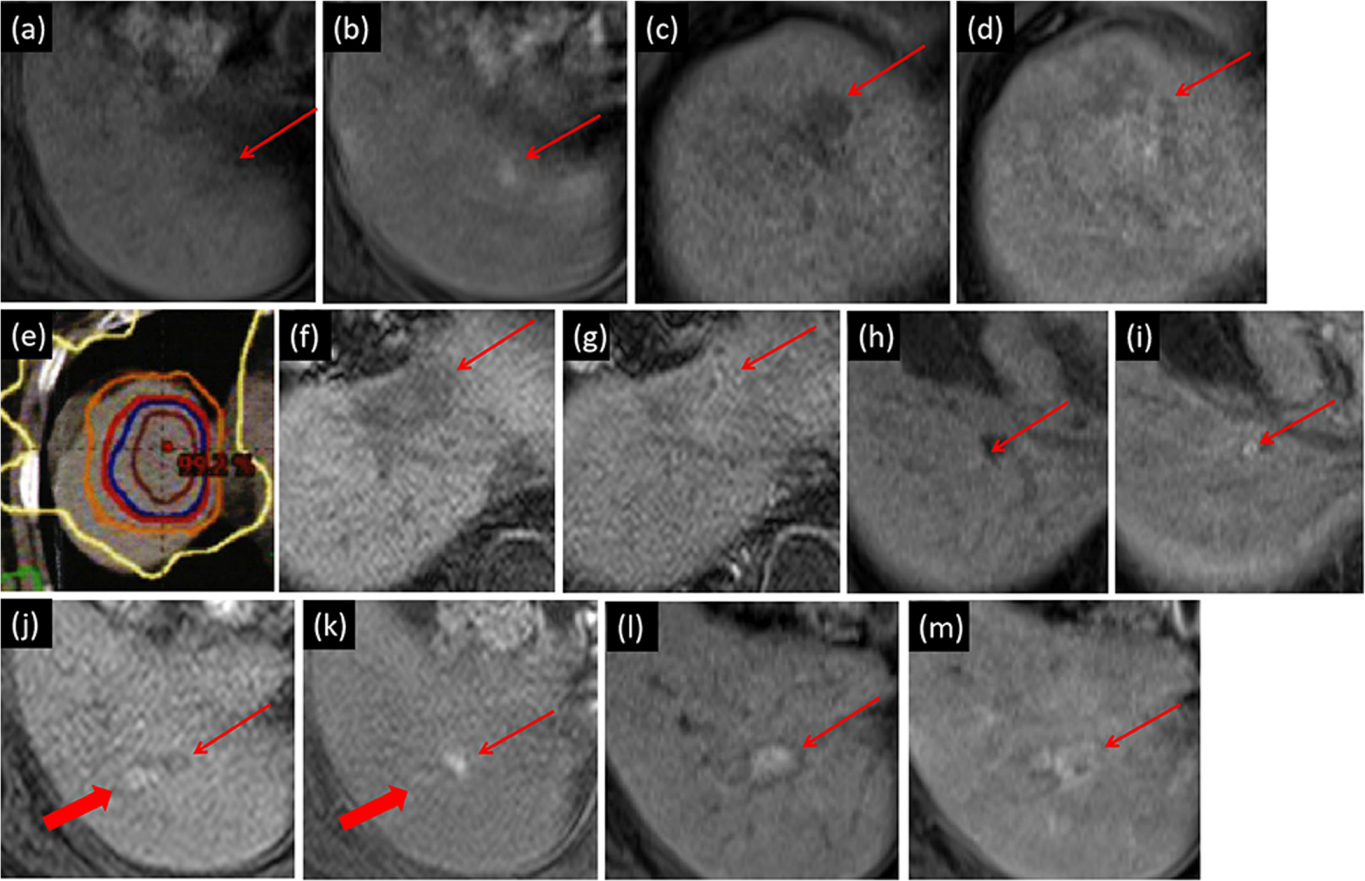
A case of progressive tumor disease and local tumor progression. (a,b) RFA was planned for an 11 mm lesion in segment (S)6, showing unenhanced T1-weighted image and AP of EOB-MRI, respectively. The lesion was hyperenhanced. (c,d) A 32 mm lesion located in S8 was visible in both the T1-weighted image (c) and the AP of the EOB-MR image (d). Because of blind areas hidden in the lung gas, RFA treatment was impossible. (e) A dose distribution picture of SBRT treatment plan was generated. As this lesion was located adjacent to the gastrointestinal tract, radiotherapy with mild hypofractionation was performed. The total dose of 42Gy (red isodose line) was delivered in 14 fractions. The central part received 60 Gy radiation. (f,g) Compared with the unenhanced T1-weighted MR image (f), at the one-year follow-up, the SBRT-treated area changed to isoenhancement (g). There was a slight liver deformation caused by radiation irritation due to SBRT treatment. (h,i) About one month after RFA treatment, in the unenhanced T1-weighted image (h) and the AP of the EOB-MR image (i), the ablation area showed scars from treatment but no appearance of recurrence. (h–k) However, a T1-weighted image was taken (h) as a reference at the 4 months’ follow-up. A hyperenhanced area, just adjacent to the initial ablated area (thick arrow, hyperenhanced scars of ablated trace), was found in the AP of the EOB-MRI (k). The local recurrence was repeatedly treated by RFA. (l,m) Two months after the second RFA treatment, the ablated area appeared as hyperenhanced scars in both the unenhanced T1-weighted image (l) and the AP of the EOB-MRI (m). Thin arrows in (a–d) and (f–m) indicate the location of the target lesion or post-treated area. This case corresponds to the No. 12 patient shown in the three tables

**Fig. 4:**
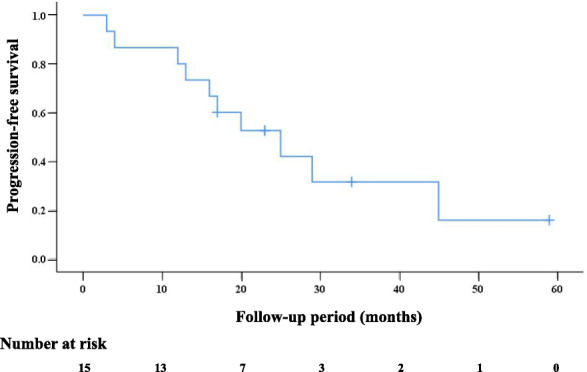
Progression-free survival (PFS) of our novel RFA and SBRT treatment strategy for 15 patients

Table 3 shows that according to mRECIST, at the end of one-year’s follow-up, the numbers (rate) of patients with one-year complete response (CR), partial response (PR), stable disease (SD), or progressive disease (PD) were 11 (73.3%), 0 (0%), 1 (6.7%), and 3 (20%), respectively (Fig. 2). Three PD cases came from one local tumor progression (patient no. 12 in Table 3, developed from incompletely-treated RFA, as shown in Fig. 3), and two intrahepatic distance recurrences occurred at 12 and 2.8 months (patients no. 3 and 14 in Table 3, respectively). According to mRECIST, as the local recurrence satisfied the criteria of more than a 20% increase in size, it was counted as PD. Because of this local recurrence at 4.2 months, the one-year local control was 97.4% (38 of 39 lesions). During the follow-up one year later, six additional intrahepatic distance recurrences (after achieving CR) and one local tumor response (developed from incompletely treated SBRT after an SD) were detected. The cumulative numbers of local tumor progression and intrahepatic distance recurrence in the whole follow-up were 2 and 8 out of 15 patients, respectively. The one- and two-year PFS rates were 80 and 52.5%, respectively (Fig. 4). The median time to progression was 20.1 (2.8–45.1) months. One female patient (no. 2) suffered from multiple intrahepatic distance recurrences and eventually died from the rapid progress of HCC due to portal vein tumor thrombus at 30 months. With a median follow-up period of 31.3 (15.1–71.9) months, the other 14 patients were still alive at the endpoint of follow-up (26 August 2020). The one- and two-year OS rates were 100% (15/15) and 89.9% (8/9), respectively.

**Table 3:** Positive and adverse effects of RFA and SBRT treatment on different multifocal lesions^1^

No.	Treatment intervals (days)^**2**^	Possible adverse reaction						Possible patient benefit			
		leukocytes	platelets	AST	ALT	T-BIL	ALBI stage	Tumor response^**3**^	Recurrence type	PFS (months)	OS (months)
1	–12	↓	N	↑	N	↑	N	CR	IDR	44.8	71.9
2	20	↓	N	N	N	N	N	CR	IDR	15.6	30.0
3	26	↓	N	N	N	N	N	PD	IDR	12.0	60.9
4	34	N	N	N	N	N	N	CR	IDR	28.7	61.2
5	20	↓	N	N	N	↑	N	CR	No	58.8	58.8
6	–4	N	N	N	N	N	N	CR	IDR	24.7	46.2
7	−11	↓	↓	N	N	N	N	CR	IDR	20.1	31.3
8	19	N	↓	N	N	N	First↓then↑	CR	No	34.1	34.3
9	45	N	N	N	N	↑	N	CR	IDR	17.1	34.3
10	48	↓	N	N	N	N	↓	SD	LTP	13.0	23.9
11	48	↓	N	N	N	N	N	CR	No	23.3	23.3
12	60	N	N	N	N	N	First↓then↑	PD	LTP	4.2	15.1
13	13	N	↓	N	N	N	N	CR	No	16.9	16.9
14	41	N	↓	↑	↑	N	First↓then↑	PD	IDR	2.8	18.5
15	55	N	N	N	N	↑	N	CR	No	22.5	22.5
Total^4^	26 (4–60)	/	/	/	/	/	4/11	11/0/1/3	2/8/5	20.1 (2.8–45.1)	31.3 (15.1–71.9)

^1^ AST: aspartate aminotransferase; ALT: alanine aminotransferase; T-BIL: total bilirubin; AFP: alpha-fetoprotein; PFS: progression-free survival; OS: overall survival; ALBI: albumin–bilirubin; CR: complete response; PR: partial response; SD: stable disease; PD: progressive disease; LTP: local tumor progression; IDR: intrahepatic distant recurrence

^2^ Here, a positive value indicates RFA was performed before SBRT, while negative values indicate that SBRT was performed before RFA

^3^ The tumor response was evaluated one year after the latter treatment (either RFA or SBRT)

^4^ In this line, the order of ALBL is abnormal and normal. The order of tumor response is CR, PR, SD, and PD. The order of recurrence type is LTP, IDR, and no recurrence. The value of treatment intervals, PFS, and OS are displayed as median and range

### Adverse effects

As shown in Table 3, in terms of the hematologic toxicity, slight (unmultiplied) reductions in leukocytes and platelet counts were found in 46.7% (7/15) and 26.7% (4/15) of patients, respectively. In terms of hepatotoxicity, the elevation rate of the ALBI grade from one week to three to five months post-treatment was 28.5% (4/15). Among them, three patients first demonstrated an increase and then a decline in ALBI grade, whereas the grade deteriorated in the remaining patient in measurements at two different time points. For the other 11 patients, the ALBI grade showed no change after treatment. After five months, abnormal values of laboratory parameters, which suggested hematologic and hepatocyte toxicity, were reversed by conservative and supportive treatment in the follow-up (data not shown). Our novel RFA and SBRT treatment exhibited no severe complications.

## Discussion

HCC cannot be completely controlled by a single treatment even using transplantation. Multi-modality treatment strategies are thus recommended [6]. So far, this study is the first to report the application of RFA and SBRT treatment for different HCC lesions coexisting in the same patients, typically as a curative strategy for patients with relatively good liver reservation (BCLC stage A4–B1). As the risks of insufficient ablation and a difficult operation are predictable, we directly planned SBRT as the foremost treatment for these lesions. Previously, SBRT was used as a salvage therapy after incomplete RFA performance [21, 23], which yielded a one- to three-year local control rate of 81.8–86.6% and an OS rate of 85.4–85.6%. Comparatively, our therapy achieved a much better treatment effect with a high one-year local control rate (97.4%), a promising prognosis with an acceptable one-year PFS (80%), and a higher OS rate (100 and 89.9% for one and two years, respectively). Given the feasibility and effectiveness of our RFA and SBRT treatment on different multifocal HCCs, this novel treatment strategy might be indicated for cases for which RFA is contraindicated.

Regarding HCCs primarily with sufficient hepatic reserve (Child–Pugh A), the reported adverse events with grade ≥ 3 for RFA treatment are mainly related to mechanical or thermal damage, including pneumothorax, pleural hemorrhage, sepsis, duodenal and colonic perforation, hepatic hemorrhage, biliary fistula, and skin burns [18, 31]. For SBRT treatment, the reported adverse events (grade ≥ 3) mainly include radiation-induced liver disease of hepatomegaly and anicteric ascites, gastrointestinal bleeding, and duodenal ulcers [18]. Before this study was conducted, we speculated that our SBRT and RFA treatment strategy may cause cumulative adverse effects because of the short treatment intervals between RFA and SBRT (4–60 days). Abdominal compression, radiation at SBRT treatment, and ablation at RFA treatment might result in anatomical deformation in both the treated zone and surrounding liver tissues [29, 32]. From this perspective, if the two lesions are close to each other, the first treatment operation (especially if the SBRT is carried out first) may change the location, shape, and texture of the lesions, thus increasing the difficulty of the later treatment. High doses of radiation stimulation from SBRT treatment cause some degree of reactive hyperemia, hepatic cell loss, hyperplasia, or parenchymal fibrosis [33]. However, unexpectedly, our novel RFA and SBRT treatment exhibited no adverse grade 3+ events. The hematologic and hepatocyte toxicities were mild and could be well controlled (reversed by conservative and supportive treatment). In this context, we find that this novel treatment strategy is safe. The adverse effects were comparable with or even lower than those of using RFA or SBRT alone (reported adverse events of grade 3+ for RFA or SBRT treatment alone are 2.6–11% and 1.6–5%, respectively [12, 18, 20]). Radiological examinations were performed every three months in the first year of follow-up, every three to six months in the second year, and every 6 − 12 months in the subsequent years depending on the clinical needs [33]. Notably, because of reactive hyperemia by high-dose radiation stimulation, AP hyperenhancement can occur after SBRT in successfully treated HCCs and may not represent a viable residual tumor. The irradiated zone after SBRT treatment usually shows as AP hyperenhancement that could be easily mistaken as a recurrence or mask a PR/CR [34]. Mendiratta-Lala et al. [35] found that 78% (39/50) of SBRT-treated lesions that had shown persistent AP hyperenhancement in MR images at three to six months gradually disappeared in the first 12 months of follow-up. Considering the possible interference of radiation-induced inflammation upon the accurate judgement of recurrence, in our study, the tumor response was evaluated at the end of one-year’s follow-up.

For HCCs less than 2 cm, the initial CR rates of RFA treatment were reported to be as high as 96% [21]. Unfortunately, in the case of multinodular or large HCC lesions, the initial CR sharply drops to almost 50% [21]. Insufficient RFA could possibly promote the proliferation of residual HCCs [36], accelerate metastasis in a variety of ways [37], and further contribute to a lower OS rate in patients with HCCs treated by RFA [38]. As RFA does not remove the corresponding hepatic segment fed by the tumor-bearing portal tributaries [39], an arterioportal fistula and intratumoral shunt develop and the intratumoral pressure suddenly increases during RFA treatment, resulting in the intravascular spread of tumor cells [38]. In this setting, the satellite foci around the target lesions possibly progress after the RFA operation. Reportedly, histopathological examinations identified satellite foci in 44% of RFA-treated HCC lesions during follow-up [40]. Our novel treatment strategy had a favorable therapeutic effect on many aspects such as CR rate, PFS rate, local control rate, and OS rate. We speculate that as the lesions treated by SBRT and RFA were located close to one another (60% lesions were in the same or adjacent segments), SBRT after RFA may inhibit the progression of satellite foci around the RFA-treated lesions, as SBRT provides 60–80% of the radiation dose to the surrounding area of the target lesions (Fig. 2). This explanation needs to be verified through in-depth research and with reliable data.

The first limitation of our study is the small number of patients and its retrospective nature. If a prospective study is planned with large sample size, the results regarding adverse effects and the prognosis might be different. Secondly, as a preliminary attempt with a novel therapy, this study was not elaborately organized with a control group and statistical analysis. The comparison of effectiveness with previously published articles may bear an underlying problem of heterogeneity between different studies. Lastly, we excluded the lesions with a non-hyperenhanced appearance in the AP of radiological examination. Most HCC-related guidelines do not allow a definitive diagnosis of HCC without AP hypervascularity (even though high-risk patients with liver cirrhosis or chronic B/C hepatitis show a hypointense appearance in the hepatobiliary phase) [41]. According to the mRECIST, evaluating viable target lesions for tumor response after locoregional treatment is only applicable to AP hypervascular/hyperenhanced lesions [30]. Therefore, unfortunately, the feasibility and effectiveness of our novel treatment strategy may not apply to non-hypervascular HCC lesions.

## Conclusions

This study is unique as we are the first to describe the feasibility of applying RFA and SBRT treatment to different multifocal lesions in one patient. The good tumor response and survival, low recurrence, and acceptable tolerance of adverse effects suggest the efficacy and feasibility of this novel treatment strategy. Nonetheless, before its application with confidence in clinical practice, a well-designed study with a large sample size and strictly selected control group must be conducted.

## Data Availability

The datasets used and/or analyzed during the current study are available from the corresponding author on reasonable request.

## Abbreviations

HCC: hepatocellular carcinoma
SBRT: stereotactic body radiotherapy
RFA: radiofrequency ablation
BCLC: Barcelona Clinical Liver Cancer
PFS: progression-free survival
OS: overall survival
ALBI: albumin–bilirubin
CR: complete response
PR: partial response
SD: stable disease
PD: progressive disease
mRECIST: modified Response Evaluation Criteria in Solid Tumors
AP: arterial phase
CT: computer tomography
MRI: magnetic resonance imaging
ALT: alanine transaminase
AST: aspartate transaminase
T-BIL: total bilirubin
ALB: albumin
